# Direct Cytotoxic and Indirect, Immune-Mediated Effects of Local Anesthetics Against Cancer

**DOI:** 10.3389/fonc.2021.821785

**Published:** 2022-01-14

**Authors:** Alejandra Wu Chuang, Oliver Kepp, Guido Kroemer, Lucillia Bezu

**Affiliations:** ^1^ Equipe Labellisée Par La Ligue Contre Le Cancer, Université de Paris, Sorbonne Université, INSERM UMR1138, Centre de Recherche des Cordeliers, Paris, France; ^2^ Metabolomics and Cell Biology Platforms, Gustave Roussy Cancer Campus, Université Paris Saclay, Villejuif, France; ^3^ Pôle de Biologie, Hôpital européen Georges Pompidou, AP-HP, Paris, France; ^4^ Service d’anesthésie, Gustave Roussy Cancer Campus, Villejuif, France

**Keywords:** local anesthetics, immunity, cancer, cell death, surgery

## Abstract

Local anesthetics are frequently employed during surgery in order to control peri- and postoperative pain. Retrospective studies have revealed an unexpected correlation between increased long-term survival and the use of local anesthetics during oncological surgery. This effect of local anesthetics might rely on direct cytotoxic effects on malignant cells or on indirect, immune-mediated effects. It is tempting to speculate, yet needs to be formally proven, that the combination of local anesthetics with oncological surgery and conventional anticancer therapy would offer an opportunity to control residual cancer cells. This review summarizes findings from fundamental research together with clinical data on the use of local anesthetics as anticancer standalone drugs or their combination with conventional treatments. We suggest that a better comprehension of the anticancer effects of local anesthetics at the preclinical and clinical levels may broadly improve the surgical treatment of cancer.

## Introduction

Malignant disease remains the second cause of death worldwide. According to the World Health Organization, cancers were responsible for 10 million deaths in 2020 (1). In most cases, treatment of solid cancers relies on tumor removal by surgical excision combined with conventional therapies such as chemotherapy and radiotherapy (2). However, standard oncological surgery may promote recurrence by facilitating cancer cell dissemination due to the mechanical removal of the tumor accompanied by the stimulation of vascular endothelial growth factor (VEGF) production by the surrounding tissue (3). Moreover, surgery often induces a stress response composed of organismal metabolic changes, local inflammation and pain, thus causing an elevation of circulating glucocorticoids and compromising antitumor immune responses (4–6). Finally, surgery negatively impacts on natural killer (NK) lymphocytes that spontaneously recognize and kill cancer cells and are known to play a determinant role in controlling tumor metastasis (7). Thus, we need novel adjuvant treatments during oncological surgery to optimally control pain, while limiting inflammation in order to decrease glucocorticoid stress, sustain anticancer immune responses and control residual cancer cells.

Surprisingly, several observational retrospective studies reported an improved overall survival after the use of local anesthetics (LAs) employed alone or in combination with general anesthesia during solid tumor resection. Thus, as compared to general anesthesia alone, the combination of epidural and general anesthesia, which is usually performed to relief major surgery-induced pain, was associated with a better long-term survival after abdominal and gynecological debulking (8–11). An enhancement of clinical progression-free time was also noticed after regional anesthesia after prostate, liver or breast primary tumor removal (12–14). Despite supplemental meta-analyses strengthening these positive outcomes, no guidelines emerged from these studies given their limits and weaknesses (15–18). However, rational hypotheses to explain these observations appeared in the literature, supporting the possibility of novel guidelines in oncological anesthesia.

Here we aim at discussing the main signaling pathways underlying the antitumor effect of local anesthetics. For this, we summarize published fundamental and clinical research while focusing on the mechanisms through which the immune system is activated by local anesthetics. We specifically dwell on their capacity to potentiate conventional antineoplastic therapies, hoping to improve clinical praxis in this area of oncology.

## Local Anesthetics Possess Direct Antitumoral Activities

### Local Anesthetics Counteract Tumor Cell Migration

**Figure 1:**
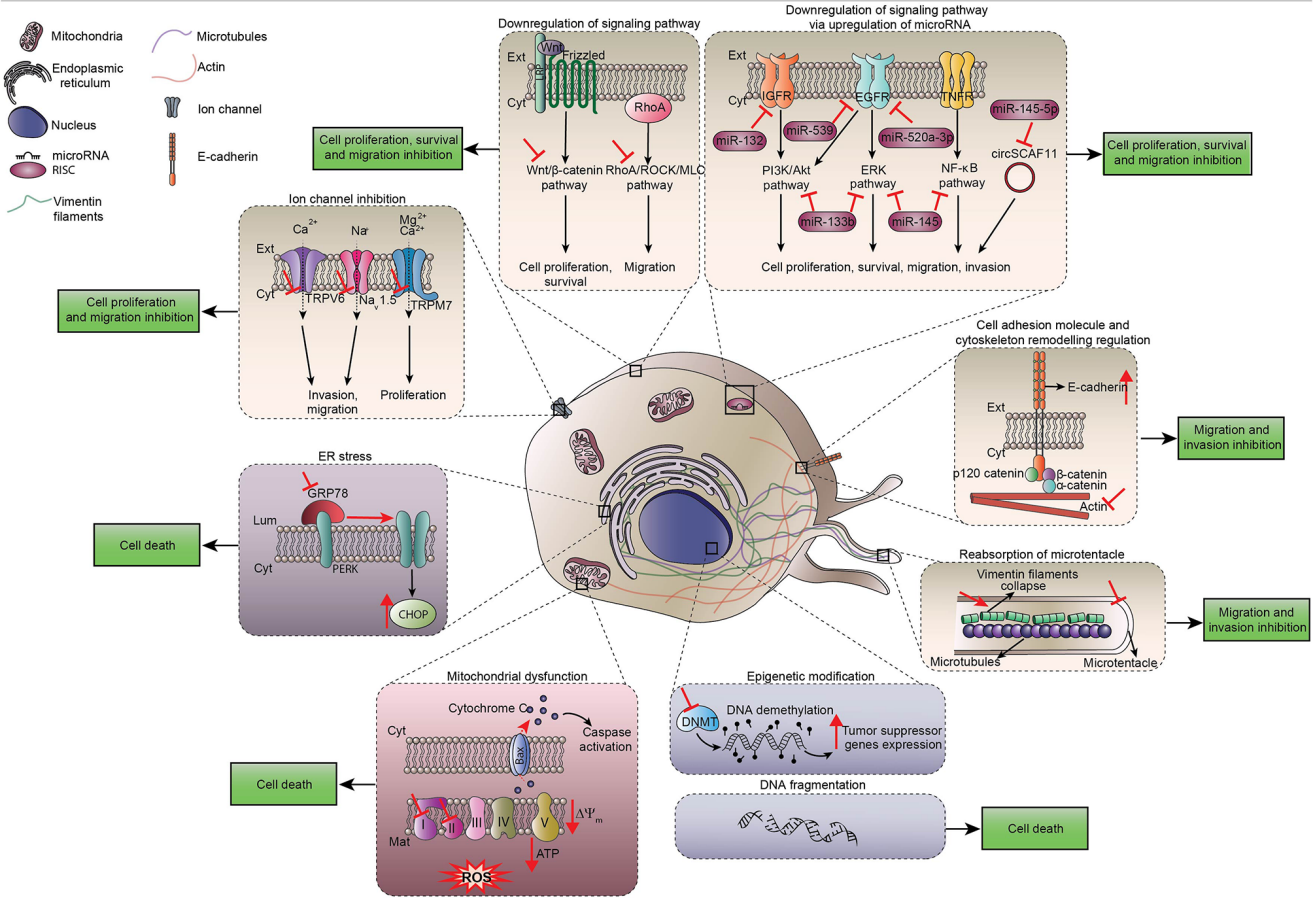
Direct cytotoxic effects of local anesthetics. Scheme summarizing direct effects of local anesthetics on cancer cells including the regulation of signaling pathways that control proliferation, survival and migration of cancer cells. Ca2+, calcium ion; CHOP, C/EBP Homologous protein; Cyt, Cytoplasm; DNMT, DNA methyltransferase; EGFR, epidermal growth factor receptor; ER, endoplasmic reticulum; Ext, extracellular space; IGFR, insulin growth factor receptor; Mg2+, magnesium ion; Na+, sodium ion; TNFR, tumor necrosis factor receptor; ROS, reactive oxygen species; PERK, protein kinase R-like ER kinase.

LAs such as lidocaine, ropivacaine, levobupivacaine, bupivacaine, procaine or chloroprocaine are used in clinical practice for their analgesic properties, which are explained by the blockade of voltage-gated sodium channels necessary for pain nerve conduction (19). Surprisingly, many observational preclinical studies noticed unexpected side effects of LAs on tumor cells. For instance, migration of cancer cells was profoundly impaired after LA exposure, likely due to effects on Ca^2+^ signaling that affect the cytoskeleton. In human triple- negative breast cancer MDA-MB-231 cells, lidocaine (10 µM or 100 µM) inhibited the CXCR4-induced Ca^2+^ release, leading to actin polymerization and impaired cytoskeletal remodeling (20). Lidocaine-inhibited migration and invasion are also mediated by TRPV6 downregulation that reduced Ca^2+^ influx in MDA-MB-231 cells, prostate cancer PC-3 cells and ovarian cancer ES-2 cells (21). Finally, infiltration of lidocaine at surgical concentrations (5-20 mM) reduced cellular migration by inhibiting the shedding of heparin-binding epidermal growth factor-like growth factor from human fibrosarcoma cells and by modulating intracellular Ca^2+^ (22). Ropivacaine was also described to increase E-cadherin protein expression and to downregulate vimentin, which is a major intermediate filament, thus contributing to reduce metastases (23). Note that tetracaine inhibits the formation of tubulin microtentacles that are required to promote reattachment of detached breast tumor cells during metastatic dissemination (24). Taken together, these findings indicate the existence of multiple molecular mechanisms by which LAs inhibit cancer cell dissemination. It is important to point out that, despite the presence of voltage-gated sodium channels on various cancer types such as breast, colon and lung tumor cells, most of the LA-induced anti-metastatic processes may be ascribed to mechanisms that do not require the inhibition of voltage-gated sodium-channels (22, 25–27) **Figure 1**.

In addition, bupivacaine, procaine and ropivacaine are endowed with the capacity to minimize the migration of neoplastic cells by inhibiting mitochondrial function. Indeed, due to their capacity to block signaling pathways operating downstream of RhoA such as the ROCK/MLC, ERK/MAPK/FAK and Rac1/JNK/paxillin/FAK pathways that commonly lead to apoptosis, local anesthetics inhibit the migration of cancer cells (25–28).

A non-negligible role of microRNAs in cancer regulation and cells migration was suggested in different models of solid cancers treated by LAs. Thus, ropivacaine enhances miT-520a-3p expression in gastric cancer cells, thereby inactivating WEE1 and PI3K/AKT signaling and inhibiting cell migration (29). Lidocaine showed an unexpected ability to up-regulate miR-145 and miR-539 expression in gastric carcinoma MKN45 cells and in lung cancer cells, respectively. These microRNAs directly downregulate epithelial growth factor receptor (EGFR), which is a prominent target for anticancer drugs and plays a major role in tumorigenesis and cancer cell invasion (30, 31). In addition, procaine induces similar antiproliferative effects by up-regulating miR-133b (32).

At clinically relevant concentrations, both lidocaine and ropivacaine block cell invasion. LAs interact with the secretion of matrix metalloproteinases (MMP) such as MMP-2 and with tumor necrosis factor (TNF) α-dependent MMP-9 involved in invasion process by inhibiting Src-dependent inflammatory signaling pathways (33, 34). This anti-invasive effect does not result from direct effects on the cytoskeleton but rather from the capacity of LAs to block cancer cell migration secondary to their anti-inflammatory properties. Indeed, Src protein tyrosine kinase plays a key role in the homeostasis of the endothelial barrier. Its activation by phosphorylation is induced in response to inflammation. Furthermore, surgical procedures provoke acute inflammatory process including vasodilatation, edema and loss of endothelial barrier integrity, thereby facilitating transmigration, extravasation and dissemination of tumor cells through lymphatic and vascular circulation **Figure 1**.

Interestingly, some LAs (lidocaine and bupivacaine) elicit an anti-invasive property at concentrations lower than those used in clinical practice (< 1mM) (21, 25). We may hypothesize that low plasma concentrations of LAs from patients receiving local or regional injection of LAs could suffice to exert systemic effects on residual cancer cells, stopping their migration.

Finally, in models of tumor resection established in immunocompetent mice that have developed syngeneic transplantable EL4 lymphomas or 4T1 breast cancers, lidocaine and bupivacaine used alone or combined with general anesthesia significantly decreased spontaneous metastasis independently of the route of administration (intravenous, spinal block or local infiltration of the inoculation site) (35–38). The mechanisms accounting for these antimetastatic effects remain unclear. However, an LA-induced reduction of circulating MMP-2 levels might contribute to impair tumor cell migration (38).

### Local Anesthetics Inhibit Tumor Cell Proliferation

LAs are able to stop tumor cell proliferation as indicated by the decrease in the mitotic marker Ki-67 as well as by a cell cycle arrest (39, 40). Most of the published data showed that this effect is concentration and time dependent (41–43). Many mechanisms may explain this process. LAs directly interfere with the advancement of the cell cycle by reducing cyclins (A2, B1, B2, D, E) and cyclin-dependent kinases expression in various models of human solid cancers (colon, lung, melanoma, thyroid, liver, breast) (28, 34, 39, 44–47). In addition, LAs induce mitochondrial dysfunction causing inhibition of respiratory chain activity and ATP production as well as a shutdown of glycolysis. This LA-induced disruption leads to mitochondrial membrane depolarization, the release of cytochrome c into the cytosol favoring the activation of apoptotic caspases, as well as cell damage mediated by reactive oxygen species (ROS) (48–51). Some LAs affect the DNA methylation status by modulating DNA methyltransferases (DNMT) activation in several types of cancer cell lines. The decrease in global methylation induced by LAs may restore the expression of previously silenced tumor suppressor genes and mediate growth-inhibitory effects on cancer cells (40, 52–58). Furthermore, some experiments suggest the implication of microRNAs in the inhibition of cancer cell proliferation (23, 29, 59). Finally, in a model of human colorectal cancer, bupivacaine and its levorotatory enantiomer levobupivacaine promote the expression of C/EBP homologous protein (CHOP), which is one of the key effectors of the endoplasmic reticulum stress response (60).

### Local Anesthetics Promote Cancer Cell Death

Many preclinical studies suggested the capacity of LAs to induce apoptosis after triggering the activation of tumor suppressor protein p53 (TP53) (61), DNA damage (62), dissipation of the mitochondrial transmembrane potential (48, 51, 63, 64), ROS production (51, 64, 65) or activation of the mitogen-activated protein kinase (MAPK) pathway (64). LAs can provoke mitochondrial rupture and cause the release of pro-apoptotic molecules such as cytochrome c (48, 63, 64) and SMAC (61). In addition, LAs upregulate the pro-apoptotic proteins Bax, Bak (31, 34, 42, 43, 47, 55, 64, 66) and down-regulate their antagonist BCL-2 (34, 42, 63, 64, 66). This ultimately favors the formation of the apoptosome (composed by APAF1, caspase 9 and cytochrome c) (67) and the proteolytic activation of a range of pro-caspases (30, 34, 51, 61–64, 68) including pro-caspase 3 (31, 34, 42, 47, 48, 51, 63, 64, 66, 69–71) and *in fine* the cleavage of poly (ADP-ribose) polymerase 1, marking the apoptotic death of cancer cells (31, 51, 63, 64, 66, 67, 71).

## Local Anesthetics May Possess Indirect Antitumoral Effects by Sustaining the Immune System

**Figure 2:**
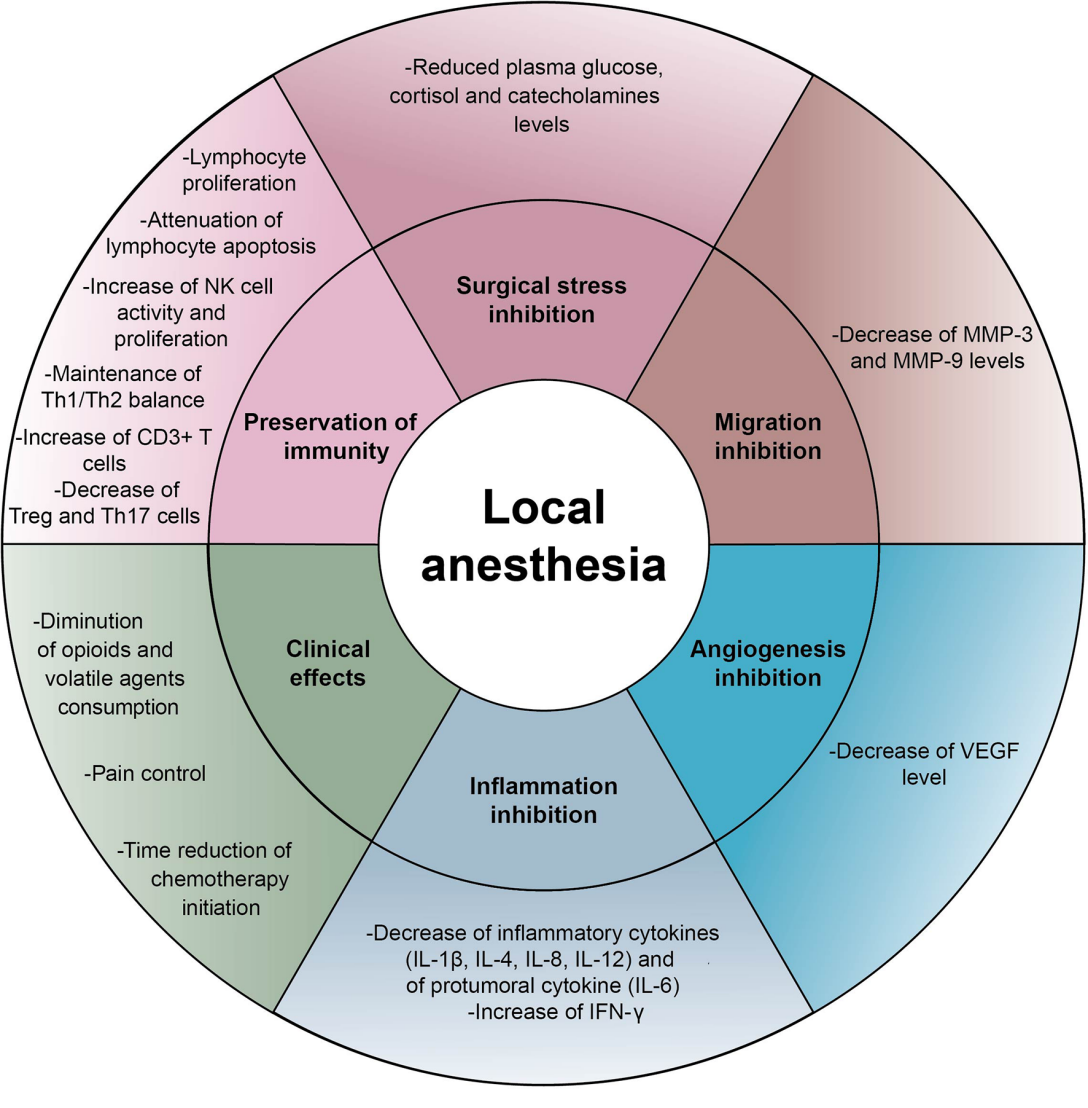
Indirect effects of local anesthetics Schematic representation of indirect effects induced by local anesthetics on cancer cells and immune effectors: inhibition of inflammation, inhibition of cancer cell proliferation and migration, surgical stress control, reduction of neoangiogenesis, preservation of immunity and clinical effects. IFN, interferon; IL, interleukin; MMP, matrix metalloproteinase; NK, natural killer cell; VEGF, vascular endothelial growth factor.

Surgery *per se* induces stress responses involving endocrine and metabolic reactions which generate acute inflammation and interact with the immune system (6). From incision, afferent nerve pathways stimulate catecholamine production and activate the corticotropic axis (6). The increase of plasma cortisol and catecholamine levels modifies the distribution of circulating leukocytes leading to lymphopenia and promotes the synthesis of the pro-tumoral cytokine IL-6, hence potentially enhancing tumor progression. Epinephrine and norepinephrine may act on beta-adrenergic receptors found in several tumor types such as breast, prostate or liver cancer and stimulate cancer cell proliferation and migration (72, 73). The adrenocorticotropic hormone (ACTH) interferes with antibody synthesis and inhibits the production of interferon (IFN) by T cells (74). This glucocorticoid stress is sufficient to profoundly subvert anticancer immunosurveillance in a range of murine models (4). In this context, it appears important to note that regional anesthesia by LAs injected into the epidural space provides a stable pain relief by blocking nociceptive pathways. Moreover, different neuroaxial anesthetic modalities possess the outstanding capacity to minimize glucocorticoid stress during surgery and to counteract the immunodepression induced by general anesthesia. Assessment of cortisol, epinephrine and norepinephrine in the serum and in the urine of patients after laparotomy under spinal anesthesia were significantly decreased during peri- and postoperative period compared to patients under general anesthesia (75–78). Thus, LAs could prevent the neuroendocrine stress responses resulting from oncological surgery and sustain anticancer immunity. This is strongly suggested by a preclinical study of Bar-Yosef et al., in which spinal block using bupivacaine not only controlled pain in rats during laparotomy but also attenuated the post-surgical dissemination of metastases (79) **Figure 2**.

Acute pain generated by surgery also compromises NK cell-mediated immunity, which is in the first line of defense against tumor development (80, 81), and fosters T helper lymphocyte polarization towards a Th2 profile (82). These findings highlight the need for optimal perioperative analgesia and the necessity to strengthen the immune system. Of note, at clinically relevant concentrations lidocaine enhances the cytotoxic effect of NK cells assessed by the release of lytic granules (granzyme B and perforin) (83). In addition, the serum from patients receiving LAs during tumor resection (independently of the route of administration) was particularly competent to kill cancer cells (84, 85), to preserve lymphocyte proliferation and to attenuate apoptosis of peripheral blood lymphocytes. The ratio of Th1/Th2 cells inclined towards a Th1 profile with secretion of IFN-γ (86). Finally, the level of Th17 and regulatory T cells (Tregs) was also significantly lower compared to the control group (87) **Table 1** and **Figure 2**.

**Table 1:** Trials assessing local anesthetics on biological markers.

Cancer	Patients	Design	Biological markers outcome	Ref
Breast	N=17	Control group: general anesthesia (sevoflurane)+opioid	PVB decreased IL-1β, MMP-3, MMP-9 and increased IL-10	(88)
	N=15	Studied group: general anesthesia (propofol) + PVB		
Breast	N=20	Studied group: general anesthesia (propofol) + PVB (bupivacaine)	PVB decreased IL-6, increased IL-12, IFN-γ and IL-10/IFN-γ ratio	(89)
	N=20	Control group: general anesthesia (sevoflurane) + fentanyl		
Breast	N=15	Control group: general anesthesia (sevoflurane)	-PVB inhibited surgical stress response (reduced plasma glucose, cortisol and C-reactive protein levels)	(90)
		Postoperative: PCA (morphine)	-No significant difference in VEGF and PGE2 values between groups	
	N=15	Studied group: general anesthesia (sevoflurane) + PVB (bupivacaine)		
Breast	N=20	Control group: general anesthesia (sevoflurane)	Increased VEGF after surgery in the general anesthesia group	(91)
		Postoperative (morphine)	TGF-β1 increased after surgery in the propofol-PVB group	
	N=20	Studied group: general anesthesia (propofol) + PVB (levobupivacaine bolus and infusion for 48h)		
Cervical	N=15	Control group: general anesthesia (sevoflurane) + fentanyl	Lidocaine preserved lymphocyte proliferation, attenuated apoptosis of peripheral blood lymphocyte, maintained the balance of Th1/Th2 cells and decreased production of cytokines	(86)
	N=15	Studied group: general anesthesia (sevoflurane) + fentanyl + bolus and infusion of lidocaine		
Colon	N=20	Control group: general anesthesia (desflurane) + epidural (ropivacaine + morphine)	Lidocaine *via* both epidural and IV routes decreased opioid consumption and reduced production of pro-inflammatory cytokines (IL-6, IL-8 and IL-1)	(92)
	N=20	Studied group: general anesthesia+ epidural analgesia (lidocaine bolus and infusion) + Postoperative epidural (ropivacaine + morphine)		
	N=20	Studied group: general anesthesia + epidural analgesia (lidocaine bolus and infusion) + lidocaine IV + Postoperative epidural (ropivacaine + morphine)		
ENT	N=15	Control group: general anesthesia (isoflurane) + morphine	Epidural analgesia decreased the requirement of morphine and stress response (blood glucose and serum cortisol)	(78)
	N=15	Studied group: general anesthesia (isoflurane) + epidural (ropivacaine)		
Liver	N=30	Control group: general anesthesia (sevoflurane)	Epidural shifted Th1/Th2 balance (Th1 dominance) and decreased Th17 and Treg cells	(87)
		Postoperative: morphine		
	N=31	Studied group: general anesthesia (sevoflurane) + epidural (bupivacaine); Postoperative: bupivacaine + morphine		
Ovary	N=30	Control group: general anesthesia (propofol) + fentanyl	Epidural group: higher NK cell cytotoxicity, higher serum concentrations of IL-10 and IFN-γ and lower serum concentrations of IL-1β and IL-8	(85)
	N=31	Studied group: general anesthesia (propofol) + fentanyl + epidural (ropivacaine + lidocaine bolus and infusion)		
Ovary	N=20	Control group: general anesthesia (volatile agents)	Intraperitoneal ropivacaine reduced time of chemotherapy initiation	(93)
	N=20	Studied group: general anesthesia (volatile agents) + intraperitoneal ropivacaine		

ENT, ear nose throat; IL, interleukin; IV, intravenous; MMP, metalloproteinase; NK, natural killer; PCA, patient-controlled analgesia; PGE2, prostaglandin E2; PVB, paravertebral block; TGF, tumor growth factor; VEGF, vascular endothelial growth factor.

Another hypothesis that might explain indirect anticancer effects of LAs is their capacity to blunt surgical inflammation. Despite the employment of minimally surgical procedures, the production of pro-inflammatory cytokines (IL-1β, IL-6 and TNF-α) and the inhibition of IFN-γ responses occur from the incision of the patient’s skin (82). Inflammation is marked by major vascular and exudative phenomena (edema, diapedesis and congestion) compromising the endothelial barrier and thus facilitating the formation of new metastases. Secretion of inflammatory cytokines also stimulates MMP-9 and VEGF production in the tumor-surrounding tissue and activates Src kinase that compromises vessel barrier integrity and facilitates cancer cell migration through the extracellular matrix (94). Moreover, the cytokine IL-6 produced in the microenvironment exerts a pro-tumor activity (95). IL-6 directly stimulates the proliferation and survival of cancer cells by stimulating the advancement of the cell cycle, the expression of anti-apoptotic molecules and angiogenesis (72, 96). In addition, IL-6 exerts immunosuppressive effects by inhibiting dendritic cells and lymphocytes, by activating Tregs and *in fine* by promoting tumor immune escape. In clinical practice, high levels of IL-6 predict chemotherapy resistance and poor prognosis in many type of cancers (97). Taken together, these data suggest that the anti-inflammatory effects of LAs may contribute to sustain immune effectors and to reduce tumor progression. Indeed, several randomized controlled trials showed a significant decrease of IL-1, IL-6, IL-8 and MMP-3 and-9 in the serum of patients after LA injection (88, 89, 92). Unfortunately, the impact on clinical outcomes has not yet been investigated **Table 1** and **Figure 2**.

## Local Anesthetics Could Impact on Oncological Outcomes

### Local Anesthetics Potentiate Conventional Anticancer Treatments

Primary tumor resection is often combined with neo-adjuvant or adjuvant anticancer treatments (chemotherapy, radiotherapy or immunotherapy) shortly before or after the surgical procedure. Interestingly, LAs can sensitize cancer cells to conventional antitumor therapeutics. Thus, the cytotoxic effects of chemotherapy (with 5-fluorouracil, paclitaxel, cisplatin or carboplatin) or protein kinase inhibitors (such as vemurafenib or erlotinib) were significantly potentiated by LAs (25, 27, 50, 54, 58, 68, 98, 99). Associated with 5-aza-2′-deoxycytidine, lidocaine showed additive demethylating effects in breast cancer cells (57). *In vivo*, the combination of cisplatin and LAs increased life span and cure rate in several mouse models (42, 100, 101), contrasting with the observation that bosutinib reversed the anti-metastatic effect of lidocaine (38). Surprisingly, procaine demonstrated an unexpected protection against cisplatin-induced nephrotoxicity as indicated by reduced blood urea nitrogen and renal tubular degeneration (102).

### Local Anesthetics Improve Overall Survival After Cancer Surgery

Many retrospective clinical studies investigated the impact of LAs on oncological prognosis. Thirteen trials suggest a potential benefit of LA injection on recurrence free survival and overall survival after cancer surgery compared to control groups. For instance, in a cohort of 588 patients undergoing primary colon cancer resection, epidural anesthesia improved the five-year survival after adjustement for relevant patient characteristics, tumor type, and type of treatment ([adjusted HR]=1.30 95% CI 1.05-1.59, p=0.01) (8). In the study of Cummings et al. involving 42 151 patients, the use of neuroaxial anesthesia significantly improved overall survival ([adjusted HR] = 0.91, 95% CI 0.87-0.94, p<0.001) (103). After hepatic resection for colorectal metastases, epidural analgesia appeared as an independent predictor of longer recurrence-free survival [HR] = 0.74, 95% CI 0.56-0.95, p=0.036) (104). After gastro-esophageal resection, epidural anesthesia increased the time to recurrence ([HR] = 0.33, 95% CI 0.17-0.63, p < 0.0001), and overall survival ([HR] = 0.42, 95% CI 0.21-0.83, p < 0.0001) at 2 years of follow-up (105). It should be noted that ten retrospective trials failed to confirm these findings. However, the putative anticancer effects of LAs are difficult to demonstate as they are influenced by various independent factors such as- cancer type, comorbidities, the drug used for local anesthesia and its posology (concentration, exposure time, administration route), as well as the combination with other anesthetics (opioids, volatile agents), which may affect immunosurveillance as well **Table 2**.

**Table 2:** Retrospective studies assessing local anesthetics impact on cancer prognosis.

Cancer	Patients	Design	Cancer prognosis outcome	Ref
Breast	N=79	Control group: general anesthesia (sevoflurane)	Studied group: lower recurrence- and metastasis-free survival (p=0.012)	(14)
		Postoperative: PCA (morphine)		
	N=50	Studied group: general anesthesia (sevoflurane) + PVB (bolus and infusion of levobupivacaine for 48h)		
Cervical	N=69	Control group: general anesthesia	Studied group: not associated with lower cancer burden or a reduced risk of tumor recurrence and mortality	(106)
	N=63	Studied group: neuraxial anaesthesia (spinal and epidural analgesia)		
Colon	N=2 299	Control group: general anesthesia + opioid-based analgesia	No association between epidural analgesia and recurrence or death	(107)
	N=449	Studied group: loading dose of lidocaine + general anesthesia and epidural anesthesia (bupivacaine with or without fentanyl for 48-72h)		
Colon	N=668	Control group: general anesthesia	Peridural analgesia:not associated with better oncological outcome	(108)
	N=208	Studied group: epidural anesthesia		
Colon	N=189	Control group: general anesthesia	Epidural analgesia: better 5-year survival (p=0.01)	(8)
	N=399	Studied group: epidural anesthesia		
Colon	N=253	Control group: general anesthesia	Epidural: lower cancer recurrence in patients older than 64 years	(109)
	N=256	Studied group: epidural anesthesia		
Colon	N=32 481	Control group: general anesthesia	Epidural anesthesia: improved survival (p<0.001)	(103)
	N=9 670	Studied group: epidural anesthesia		
Colo-rectal	N=93	Control group: general anesthesia sevoflurane or desflurane + fentanyl and IV morphine for 2 to 5 days	Epidural anesthesia: lower mortality in the sub-group of rectal cancer (p=0.049)	(110)
	N=562	Studied group: general anesthesia sevoflurane or desflurane + epidural (bolus local anesthetic and fentanyl or local anesthetic alone and infusion of local anesthetic with fentanyl or local anesthetic and morphine for 2-5 days)		
Colo-rectal	N=173	Control group: PCA (morphine)	No significant difference in overall survival or disease-free survival at 5 years	(111)
	N=107	Studied group: epidural anesthesia (Bolus and infusion of bupivacaine with fentanyl for 48h)		
	N=144	Studied group: spinal anesthesia (bupivacaine with morphine)		
Colo-rectal	N=307	Control group: general anesthesia (isoflurane or desflurane + fentanyl)	Epidural analgesia: greater long-term survival (p<0.02)	(9)
	N=442	Studied group: general anesthesia (isoflurane or desflurane + fentanyl) + epidural analgesia		
Colo-rectal + liver metastases	N=120	Control group: IV anesthesia	Epidural anesthesia: improved five-year recurrence free survival (p=0.036)	(104)
	N=390	Studied group: epidural anesthesia		
Gastro-oeso-phageal	N=140 (total)	Control group: general anesthesia (sevoflurane or propofol infusion) + IV opioid analgesia	Epidural was associated with 2-year recurrence and overall survival benefit (p<0.0001)	(105)
		Studied group: general anesthesia (sevoflurane or propofol) + epidural anesthesia (bupivacaine bolus + infusion with morphine for 96h)		
ENT	N=160	Control group: general anesthesia + morphine	Epidural anesthesia:increased cancer-free survival (p=0.04) and overall survival (p=0.03)	(112)
	N=111	Studied group: general anesthesia + epidural anesthesia		
Liver	N=244	Control group: general anesthesia (sevoflurane or propofol) + sufentanil + nonsteroidal anti-inflammatory drugs	Local anesthetic increased recurrence free survival (p=0.002) and overall survival (p=0.036)	(12)
	N=245	Studied group: lidocaine+nonsteroidal anti-inflammatory drugs		
Melanoma	N=221	Control group: general anesthesia (isoflurane or propofol) + sufentanil or remifentanil	Spinal anesthesia: a trend of better cumulative survival rate	(113)
	N=52	Studied group: spinal anesthesia (bupivacaine)		
NSCLC	NA	Control group: general anestheisa (isoflurane, sevoflurane or desflurane) + IV opioid analgesia; postoperative PCA (hydromorphone, fentanyl or morphine)	No difference on recurrence-free survival or overall survival	(114)
		Studied group: general anesthesia (isoflurane, sevoflurane or desflurane) + IV opioid analgesia		
		Postoperative: epidural (bupivacaine + fentanyl or bupivacaine + hydromorphone or ropivacaine and fentanyl)		
		Studied group: general anesthesia (isoflurane, sevoflurane, or desflurane) + IV opioid analgesia		
		Postoperative: epidural/PCA: bupivacaine + fentanyl or bupivacaine + hydromorphone or ropivacaine + fentanyl		
Ovary	N=37	Control group: general anesthesia (sevoflurane or isoflurane) + PCA fentanyl	Epidural anesthesia: greater 3- and 5-year overall survival rates (p=0.043)	(10)
	N=106	Studied group: epidural anesthesia (Infusion of bupivacaine or ropivacaine and morphine for 48h)		
Ovary	N=43	Control group: general anesthesia (volatile + fentanyl)	Epidural anesthesia: not associated with improved overall survival or time to recurrence	(115)
		Postoperative: ketorolac and PCA (morphine)		
	N=37	Studied group: general anesthesia +epidural anesthesia (bolus of bupivacaine with or without fentanyl); Postoperative: ketorolac and epidural for 48h		
Pancreas	N=2 239 (total)	Control group: general anesthesia (sevoflurane) + epidural analgesia (ropivacaine)	Lidocaine group:longer overall survival (p=0.013)	(11)
		Studied group:lidocaine bolus+ continuous infusion + general anesthesia (sevoflurane) + epidural analgesia (ropivacaine);		
Prostate	N=123	Control group: general anesthesia(propofol) + fentanyl	Epidural anesthesia: lower risk of recurrence (p=0.012)	(13)
		Postoperative: PCA (morphine)		
	N=102	Studied group: general anesthesia (propofol) + fentanyl		
		Postoperative: local anesthetic infusion for 48-72h		
Prostate	N=158	Control group: general anesthesia (isoflurane) + fentanyl; Postoperative: ketorolac + paracetamol	Epidural analgesia: improved clinical progression-free survival (p=0.002).	(116)
	N=103	Studied group: general anesthesia (isoflurane) + Epidural (bupivacaine) + fentanyl		
Prostate	N=533	Control group: intravenous analgesia	Epidural analgesia:not associated with a significant effect	(117)
	N=578	Studied group: epidural analgesia		
Visceral	N=63	Control group: general anesthesia (isoflurane + fentanyl);	A trend in favor of epidural anesthesia was observed for recurrence free survival	(118)
		Postoperative: morphine		
	N=69	Epidural group: bupivacaine + general anesthesia (isoflurane); postoperative: bupivacaine + morphine		

IV, intravenous; PCA, patient-controlled analgesia; PVB, paravertebral block.

PCA, patient-controlled analgesia; IV, intravenous.

Irrespective of these limitations, four large meta-analyses all concluded in favor of beneficial effects of epidural anesthesia alone or associated with general anesthesia. With 14 studies including 47 000 patients, Chen et al. demonstrated an improved overall survival of epidural anesthesia compared with general anesthesia alone (HR = 0.84, 95% CI 0.74-0.96, p = 0.013) (15). In the meta-analysis by Pei et al., combined general-epidural anesthesia was associated with decreased recurrence and metastasis rate in the subgroup of prostate cancer patients and in the subgroup with followup less than or equal to 2 years (OR = 0.66, 95% CI 0.46-0.95, p=0.027; OR = 0.70, 95% CI 0.51-0.98, p=0.035; respectively) (16). Sun et al. showed similar results with a significant better overall survival for patients receiving perioperative regional anesthesia ([HR] = 0.84, 95% CI, 0.75-0.94; *I* ^2^ =41%) compared to the control group (17). Finally, the meta-analysis by Weng et al. involving 21 studies and 51 620 patients concluded that neuroaxial anesthesia improved both overall survival ([HR] = 0.853, CI= 0.741-0.981, p=0.026) and recurrence-free survival ([HR] = 0.846, CI=0.718-0.998, p=0.047) (18) **Table 3**.

**Table 3:** Meta-analyses assessing local anesthetics impact on cancer prognosis.

Cancer	Patients	Design	Cancer prognosis outcome	Ref
Solid tumors	14 studies (47 000 patients)	Control group: general anesthesia	Epidural anesthesia improved overall survival (p=0.013).	(15)
		Studied group: epidural anesthesia with or without general anesthesia		
Solid tumors	10 studies (3254 patients)	Control group: general anesthesia	Combined general-epidural anesthesia was associated with decreased recurrence (p=0.027) and metastasis rate (p=0.035) within the subgroup of prostate cancer patients and the subgroup with follow-up less than or equal to 2 years	(16)
		Studied group: combined general-epidural anesthesia		
Solid tumors	20 studies (NA)	Control group: general anesthesia	Perioperative regional anesthesia associated with improved overall survival ([HR] = 0.84, 95% CI, 0.75-0.94; *I* ^2^ =41%)	(17)
		Studied group: perioperative regional anesthesia		
Solid tumors	21 studies (51 620 patients)	Control group: general anesthesia	Neuroaxial anesthesia improved overall survival (p=0.026) and recurrence-free survival (p=0.047)	(18)
		Studied group: neuroaxial anesthesia combined with or without general anesthesia		

Finally, among 11 prospective randomized controlled trials, two studies reported a better disease-free survival after epidural anesthesia (ropivacaine or bupivacaine) associated with intravenous or volatile agents during colon (p=0.012) or bladder tumor resection (p=0.02) compared to general anesthesia alone (119, 120). One study investigated the antitumor activity of patient sera after levobupivacaine infiltration during breast cancer resection. A significant blockade of MDA-MB-231 breast carcinoma cells was observed (p=0.01) (121). A better survival after hepatectomy was also noticed after infiltration of ropivacaine close to the incision site (p=0.029) (122). However, other trials failed to confirm these findings, perhaps due to a lack of power and major confusion bias compromising data analyses (injection of multiple different anesthetic agents, inclusion of cancers at different stages, loss of patients due to deficient followup, heterogenous groups…). **Table 4** Multicenter randomized controlled trials with high quality of methodology are urgently awaited to definitevely conclude on the potential benefit of LAs on oncological outcomes.

**Table 4:** Randomized controlled trials assessing local anesthetics impact on cancer prognosis.

Cancer	Patients	Design	Cancer prognosis outcome	Ref
Bladder	N=150	Control group: general anesthesia (sevoflurane)+fentanyl	Local anesthesia: longer disease-free survival (p=0.02)	(119)
		Postoperative (morphine)		
	N=510	Studied group (propofol) +lidocaine+ epidural (ropivacaine)		
Breast	N=11	Control group: general anesthesia (sevoflurane) + morphine postoperative: PCA (morphine)	Patient serum from studied group reduced MDA-MB-231 breast carcinoma cell proliferation (p=0.01)	(121)
	N=11	Studied group: general anesthesia (propofol) + PVB (bolus and infusion of levobupivacaine)		
Breast	N=30	Control group: general anesthesia (volatile anesthetic)	No difference between groups	(123)
	N=30	Studied group: general anesthesia (volatile anesthetic) + PVB (ropivacaine bolus and infusion)		
Breast	N=1065	Control group: general anesthesia (sevoflurane)	No difference between groups	(124)
	N=1043	Studied group: general anesthesia (propofol) + PVB		
Breast	N=58	Control group: general anesthesia (propofol)	No difference between groups	(125)
	N=56	Studied group: general anesthesia + single injection PVB (ropivacaine)		
	N=59	Studied group: general anesthesia + continuous-PVB (ropivacaine for 72h)		
Colon	N=92	Control group: general anesthesia (isoflurane)+ fentanyl	Epidural improved survival in patients without metastases (p=0.012)	(120)
	N=85	Studied group: general anesthesia (isoflurane) + fentanyl + epidural group (bupivacaine)		
Colon Rectum	N=30	Control group: general anesthesia (propofol+ remifentanyl); postoperative: PCA fentanyl	No difference for postoperative NK cell cytotoxicity and IL-2, recurrence or metastasis	(126)
	N=30	Studied group: general anesthesia (propofol and remifentanyl) + surgical wound infiltration of ropivacaine		
Liver	N=20	Control group: tramadol injections	Ropivacaine increased postoperative survival (p=0.029)	(122)
	N=20	Studied group: local incision analgesia (ropivacaine bolus + infiltration)		
	N=20	Studied group: PCA (fentanyl)		
Lung	N=200	Control group: general anesthesia (propofol/sevoflurane+ sufentanyl/remifentanyl); postoperative: PCA morphine	No difference between groups for recurrence-free and overall survival	(127)
	N=200	Studied group: general anesthesia (propofol/sevoflurane+ sufentanyl/remifentanyl)+ epidural anesthesia (ropivacaine)		
Prostate	N=50	Control group: general anesthesia; postoperative: morphine	No difference between groups	(128)
	N=49	Studied group: general anesthesia + ropivacaine bolus and infusion with fentanyl		
Solid tumors	N=216	Control group: general anesthesia; postoperative: opioid-based analgesia	No difference between groups	(129)
	N=230	Studied group: general anesthesia + epidural group (bupivacaine or ropivacaine); postoperative: continous bupivacaine or ropivacaine + fentanyl or pethidine		
Solid tumors	N=822	Control group: general anesthesia (propofol/sevoflurane+ sufentanyl/remifentanyl/fentanyl); postoperative: PCA morphine	No difference between groups for overall survival	(130)
	N=772	Studied group: general anesthesia (propofol/sevoflurane+ sufentanyl/remifentanyl/fentanyl)+ epidural anesthesia (ropivacaine)		

PCA, patient-controlled analgesia; NK, natural killer; PVB, paravertebral block.

Until now, no guidelines and no recommendations in onco-anesthesia are available to guide clinical practice. Indeed, most of the results issued from clinical studies are not convincing enough to elaborate new guidelines due to a lack of power, presence of bias, heterogeneity of groups and the combined use of various anesthetics that exert conflicting effects on tumor cells. However, based on the sheer number of prospective multicenter randomized controlled trials, we may expect the translation of preclinical data into the clinics for the near future. Thus, we anticipate that Phase III clinical trials will confirm that, beyond their useful analgesic properties, local anesthetics exert antitumor effects, meaning that their use will be approved for this additional indication.

## Discussion

Oncological surgery generates neuroendocrine stress, inflammation and acute pain responsible for immunosuppression, hence impacting on the antitumor immune response (4, 83). The manipulation of the tumor by the surgeon, vascular invasion and the peri-operative synthesis of VEGF also promote the migration and proliferation of residual cancer cells and thus, future metastatic recurrence (131).

The impact of local anesthetics on cancer and its recurrence after surgery has spurred a wave of interest over the last decade. Two recent reviews covering this field have been published (132, 133). In the present article we attempted to synthesize the current preclinical and clinical state of the art, while evoking the capacity of local anesthetics to stimulate anticancer immune responses, thereby potentiating the efficacy conventional anticancer therapies. Particular emphasis has been laid on the difference direct effects impacting on cancer cells and indirect, immune-mediated effects controlling residual tumor cells that mediate local relapse or distant metastasis.

LAs possess analgesic and anti-inflammatory properties that indirectly improve cancer immunosurveillance. In addition, LAs have direct molecular effects on mitochondrial metabolism, generate oxidative stress, trigger apoptosis pathways in cancer cells and activate NK cells (34, 64). Preclinical studies found that treatment of cancer cells with clinically relevant concentrations of LAs inhibits their proliferation and migration or induces cell death (39). These direct antitumor effects described in many cancer cell lines are time- and concentration-dependent. In murine models, LAs showed a remarkable ability to decrease the incidence of metastases after surgery (35, 38). In humans, several clinical studies noticed that LAs used for extradural block attenuated the immunosuppressive endocrine effects generated by surgery (75). In addition, an array of retrospective trials and meta-analyses concluded that LAs used alone or in combination with general anesthesia preserved NK cell activity and improved overall survival and recurrence-free survival (18).

Several putative mechanisms may explain the antitumor properties of LAs. First, LAs reduce the immunosuppressive effects of surgery by reducing glucocorticoid stress and by dampening inflammation (88). Second, LAs stimulate the proliferation and the activity of NK cells that play an important role in the innate immune defense against cancer (83). Third, LAs have direct toxicity on cancer cells and may induce apoptosis before residual cancer cells migrate into adjacent tissues or reach the lumen of lymphatic or vascular capillaries. Finally, LAs reduce the consumption of major protumor molecules such as opioids and volatile agents during cancer surgery (78, 92). Preclinical data sustaining these findings are rather convincing as they have been reproduced in many cancer types. However, these promising data now need translation into the clinics. The outcome of ongoing randomized multicenter prospective trials dealing with the potential anticancer effects of LAs are urgently awaited. Indeed, the confirmation that LAs improve patient outcome would have a major impact on clinical practice, in particular in the context of oncological surgery.

## Author Contributions

AW provided the list of trials and designed the figures. OK helped for the design of figures. GK and LB wrote the manuscript. All authors contributed to the article and approved the submitted version.

## Funding

OK is supported by Institut National du Cancer (INCa) and the DIM Elicit of the Ile-de-France; LB received a research grant by Bristol Myers Squibb Foundation France. AW was supported by El Programa Nacional de Becas “Don Carlos Antonio Lopez” (BECAL). GK is supported by the Ligue contre le Cancer (équipe labellisée); Agence National de la Recherche (ANR) – Projets blancs; AMMICa US23/CNRS UMS3655; Association pour la recherche sur le cancer (ARC); Association “Ruban Rose”; Cancéropôle Ile-de-France; Fondation pour la Recherche Médicale (FRM); a donation by Elior; Equipex Onco-Pheno-Screen; European Joint Programme on Rare Diseases (EJPRD); Gustave Roussy Odyssea, the European Union Horizon 2020 Projects Oncobiome and Crimson; Fondation Carrefour; INCa; Inserm (HTE); Institut Universitaire de France; LabEx Immuno-Oncology (ANR-18-IDEX-0001); the Leducq Foundation; a Cancer Research ASPIRE Award from the Mark Foundation;, the RHU Torino Lumière; Seerave Foundation; SIRIC Stratified Oncology Cell DNA Repair and Tumor Immune Elimination (SOCRATE); and SIRIC Cancer Research and Personalized Medicine (CARPEM). This study contributes to the IdEx Université de Paris ANR-18-IDEX-0001.

## Conflict of Interest

OK is scientific co-founder of Samsara Therapeutics; GK has been holding research contracts with Daiichi Sankyo, Eleor, Kaleido, Lytix Pharma, PharmaMar, Samsara, Sanofi, Sotio, Vascage and Vasculox/Tioma. GK is on the Board of Directors of the Bristol Myers Squibb Foundation France. GK is a scientific co-founder of everImmune, Samsara Therapeutics and Therafast Bio. GK is the inventor of patents covering therapeutic targeting of aging, cancer, cystic fibrosis and metabolic disorders.

The remaining authors declare that the research was conducted in the absence of any commercial or financial relationships that could be construed as a potential conflict of interest.

The funders were not involved in the study design, collection, analysis, interpretation of data, the writing of this article or the decision to submit it for publication.

## Publisher’s Note

All claims expressed in this article are solely those of the authors and do not necessarily represent those of their affiliated organizations, or those of the publisher, the editors and the reviewers. Any product that may be evaluated in this article, or claim that may be made by its manufacturer, is not guaranteed or endorsed by the publisher.

## Abbreviations

ACTH, adrenocorticotropic hormone; Ca2+, calcium ion; DNMT, DNA methyltransferase; EGA, epidural-general anesthesia; EGFR, epithelial growth factor receptor; GA, general anesthesia; HB-EGF, heparin-binding epidermal growth factor-like growth factor; IFN, interferon; IL, interleukin; LA, local anesthetic; MMP, matrix metalloproteinase; NK, natural killer; PCA, patient controlled analgesia; PGE2, prostaglandin E2; PVB, paravertebral block; ROS, reactive oxygen species; TGF, tumor growth factor; TNF-α, tumor necrosis factor α; VEGF, vascular endothelial growth factor.
